# Catalyzing Knowledge-Driven Discovery in Environmental Health Sciences through a Community-Driven Harmonized Language

**DOI:** 10.3390/ijerph18178985

**Published:** 2021-08-26

**Authors:** Stephanie D. Holmgren, Rebecca R. Boyles, Ryan D. Cronk, Christopher G. Duncan, Richard K. Kwok, Ruth M. Lunn, Kimberly C. Osborn, Anne E. Thessen, Charles P. Schmitt

**Affiliations:** 1 Office of Data Science, National Institute of Environmental Health Sciences (NIEHS), Durham, NC 27709, USA; charles.schmitt@nih.gov; 2 Research Computing, RTI International, Durham, NC 27709, USA; rboyles@rti.org; 3 Health Sciences, ICF, Durham, NC 27713, USA; ryan.cronk@icf.com; 4 Genes, Environment, and Health Branch, Division of Extramural Research and Training, NIEHS, Durham, NC 27709, USA; christopher.duncan@nih.gov; 5 Epidemiology Branch, Division of Intramural Research, NIEHS, Durham, NC 27709, USA; richard.kwok@nih.gov; 6 Office of the Director, NIEHS, Bethesda, MD 20892, USA; 7 Integrative Health Assessment Branch, Division of the National Toxicology Program, NIEHS, Durham, NC 27709, USA; lunn@niehs.nih.gov; 8 Health Sciences, ICF, Fairfax, VA 22031, USA; Kim.Osborn@icf.com; 9 Environmental and Molecular Toxicology Department, Oregon State University, Corvallis, OR 97331, USA; annethessen@gmail.com

**Keywords:** community of practice, community-driven, controlled vocabulary, metadata, taxonomy, ontology, standards, semantic, environmental health, toxicology

## Abstract

Harmonized language is critical for helping researchers to find data, collecting scientific data to facilitate comparison, and performing pooled and meta-analyses. Using standard terms to link data to knowledge systems facilitates knowledge-driven analysis, allows for the use of biomedical knowledge bases for scientific interpretation and hypothesis generation, and increasingly supports artificial intelligence (AI) and machine learning. Due to the breadth of environmental health sciences (EHS) research and the continuous evolution in scientific methods, the gaps in standard terminologies, vocabularies, ontologies, and related tools hamper the capabilities to address large-scale, complex EHS research questions that require the integration of disparate data and knowledge sources. The results of prior workshops to advance a harmonized environmental health language demonstrate that future efforts should be sustained and grounded in scientific need. We describe a community initiative whose mission was to advance integrative environmental health sciences research via the development and adoption of a harmonized language. The products, outcomes, and recommendations developed and endorsed by this community are expected to enhance data collection and management efforts for NIEHS and the EHS community, making data more findable and interoperable. This initiative will provide a community of practice space to exchange information and expertise, be a coordination hub for identifying and prioritizing activities, and a collaboration platform for the development and adoption of semantic solutions. We encourage anyone interested in advancing this mission to engage in this community.

## 1. Introduction

The use of a harmonized language to describe scientific methods and discoveries is well recognized as being critical for a variety of needs, including searching the literature, integrating data and knowledge, and conducting comparative analyses. Given the diversity of environmental health sciences research and practice, the use of a harmonized language spanning biomedical sub-disciplines and other fields (e.g., environment, climate, disaster, population health) is important. Developing and applying a common, harmonized language is an ongoing and challenging task, as it must keep pace with scientific advances that generate new methods and subsequently new data types and knowledge.

We define “environment” broadly as any external influences on health, including physical, chemical, biological, social and cultural factors, and related behaviors [1]. An EHS language covers diverse scientific sub-domains and technical and semantic fields that include: data elements collected during scientific inquiries (e.g., PhenX measures, NHANES data elements, Tox21 endpoints, Disaster Research Response (DR2) tools); terminologies and metadata that are used to describe data elements; ontologies that formalize knowledge representation, such as the Gene Ontology (GO) [2]; mappings that link data elements and metadata to ontologies; and knowledge bases that combine and harmonize terminologies and ontologies (e.g., Comparative Toxicogenomics Database [3], Monarch Initiative [4]). Language harmonization efforts should support and promote the tools that help to capture, represent, and apply vocabulary elements, such as chemical read-across models [5] and gene enrichment analysis [6]. The future of an environmental health sciences language is expected to incorporate machine-oriented language constructs that are built on linguistic-based representations [7], such as word- and concept-embeddings [8], that facilitate machine learning methods to conduct data integration and reasoning [9,10,11]. Work to integrate human-focused vocabularies with machine-focused language constructs is progressing [12,13,14] and shows promise for biomedical analysis [15,16].

We highlight and seek support for a community-based initiative to help define and address gaps in the EHS language, including terminologies, ontologies, mapping, knowledge bases, tools, and emerging means of representing knowledge. Our initial work describes the formation of community processes, which includes the development of use cases and competency questions to identify the gaps and challenge the value of using existing approaches; workshops and webinars to identify and focus activities on targeted issues; and engagement with communities that are developing and applying standardized vocabularies in subfields (e.g., earth and social sciences). We emphasize a more sustainable approach that leverages the existing governance structures and communication platforms of the Research Data Alliance (RDA) model [17] to address focused use cases that are relevant to the sharing and integration of EHS data across heterogeneous sources and modalities [17]. These improvements address challenges that have hindered similar efforts in the past.

## 2. Discussion

To frame this objective, we present representative challenge areas and recent advances, followed by efforts to lay the foundation for a sustainable EHS language community.

### 2.1. Representative Challenge Areas

*Disease Modeling*: Incorporating exposure data in disease models is challenging because of the complicated metadata that is needed to characterize the exposure [18]. Issues surrounding the temporality of exposure, such as the timing relative to a life cycle [19], duration of exposure [20], frequency of exposure [21], latency [22], route of exposure [23], and point of contact are critical metadata [24]. Another modeling concern is that environmental exposures do not occur in isolation and interactions between multiple exposures can be critical to exposure health impacts [25]. Evidence codes [26] and probability measures [27] are needed to correctly weigh a piece of evidence in an integrated data set. Metadata increases the size and complexity of the model but are essential for correctly interpreting the data. In addition to the complex cause-and-effect relationships, translating exposure data from a human-readable to a computable format is difficult because the laboratories producing these data are not using a community-wide standard [28]. Developing, maintaining, and sustaining these standards is challenging for any community and requires significant time and resources [29].

*Systems Toxicology*: The field of toxicology continues to transition from predominately animal testing to a spectrum of in vivo, in vitro, and in silico approaches, with each focused on gaining specialized knowledge on outcomes and bio-chemical mechanisms [30]. Despite often high levels of control over toxicology testing conditions, challenges persist regarding accurately and systematically describing key observations across labs [31,32,33], which can lead to significant costs to integrate observations, where quality and data loss issues occur when data are integrated. The evolving mix of testing paradigms, organisms, in vitro platforms, and assays are increasing the diversity, volume, and specificity of toxicology data. This makes integration at the observational level more challenging and increases the need to integrate and compare data and findings at the knowledge level. As such, constructing bridges between the collection of toxicology assays and descriptions of bio-chemical and bio-mechanistic processes (e.g., adverse outcome pathways (AOPs), GO-causal activity models) is increasingly important [34,35] and a challenge as newer assays continue to emerge.

*Precision Medicine*: As precision medicine transitions to practice, a critical challenge is to identify both the genetic etiologies and environmental factors in disease in order to translate basic science into prospective interventions and advance healthcare [25]. If we include gene and phenotype relationships that are derived using model organisms, approximately 83% of known human coding genes are matched with available phenotype data [4], but the environmental component of these relationships is underrepresented in public databases and knowledge bases [36]. Without considering the effect of the environment, our ability to understand human disease and realize precision health is limited, even with the promise of modern genomics [37]. Achieving the integration of phenotype, genotype, and environmental information (including psychosocial stressors and cultural factors) requires an extensive translation of data into a computable form and the extension of the gene/phenotype data model, which are both done to ensure the discovery of extant data and to provide a structure that encourages new data discoveries and analyses. The informatics approaches that are needed to address these challenges have historically been focused on genomics, with less attention paid to additional types of data streams. Consequently, the types of algorithms that are used for genetic diagnostics are not accessible for diseases that have critical environmental components [38], such as a spectrum of environmental causes, exacerbations, compensatory mechanisms, repair, and potential therapeutic interventions.

### 2.2. Recent Efforts

Biomedical knowledge bases integrate information that is contained in terminologies, ontologies, and literature, providing great potential to find patterns in data that are hidden due to the volume, heterogeneity, and complexity of that data [39]. They are important resources for supporting the interpretation of findings and hypothesis generation by researchers. Efforts to integrate biomedical databases have resulted in the creation of several related, but often unconnected, biomedical knowledge graphs [40]. While these graphs are being used to better understand cancer [41], identify new drug candidates [42], and diagnose rare diseases [43], insights about the effect of environmental exposures are not forthcoming, largely because the environmental aspect of diseases is not included in these knowledge graphs. This is for two reasons: there are few curated data sources that associate environmental exposures to phenotypic outcomes in a structured manner [18,44], and the complexity of exposure science has not yet been modeled sufficiently using modern semantic structures to allow for large-scale data integration [18].

Development of the standards for associating environmental exposures to phenotypic outcomes and the associated metadata is a vast undertaking that requires substantial community engagement. The Comparative Toxicogenomics Database (CTD) has made substantial progress in aggregating and standardizing the associations between exposures, chemicals, genes, and diseases. Its exposure ontology (ExO) provides a semantic model of an exposure event and its outcome [3,45]. The Environmental Conditions, Treatments, and Exposures Ontology (ECTO) was developed based on the ExO to model exposure events for use in semantic models of disease, and the ENVO environmental ontology [46] provides linkages to environmental entities and processes. Several knowledge bases, such as the NCATS Translator [47], Monarch Initiative [48], and PheKnowLator [49], are incorporating environmental and chemical exposures from sources, such as the CTD and AOP Wiki [50], and linking that information to other knowledge constructs (e.g., diseases, phenotypes, genes, variants, therapeutics). A European strategy for exposure science has been proposed that includes substantial community building and data integration infrastructure development [51].

Relatedly, there have been promising advances in the development of data and metadata standards and the mapping of data elements to ontologies. Efforts within the field of toxicology, including UMLS and Adverse Outcome Pathways (AOPs), map tox-related assays to standards [52]. The Children’s Health Exposure Analysis Resource (CHEAR) and Human Health Exposure Analysis Resource (HHEAR) programs have developed an interdisciplinary CHEAR/HHEAR ontology that supports the analysis of the exposome through the harmonization of health and exposure data that is consistent across the program [53,54,55]. The NIEHS Superfund Basic Research and Training Program (SRP) has supported efforts to foster data sharing, interoperability, and reuse through the broader adoption of data standards and ontologies to support cross-center research collaborations [56]. These efforts help to advance cross-center standards and support for minimal information standards in the environmental health sciences domain, such as MIATE (Minimum Information about Animal Toxicology Experiments (in vivo) 2021). The National Institutes of Health (NIH) has increased focus on generating and adopting common data elements (CDEs), such as those in the PhenX Toolkit and the NLM Common Data Elements (CDE) Repository. Recent CDE development has been directed at supporting COVID-19 research through the Disaster Research Response (DR2) portal and their work with the RADx initiatives [57,58].

Despite these advances, more work is needed, particularly in implementing the sociological aspects of community-driven standards development. Several workshops have been held to mobilize the EHS community around standards development [59,60] and a strategy for toxicology ontology development was proposed [61]. The exposure science community is exploring the use of ontologies in research, both for data integration and gaining new insights [28,50,62,63,64,65,66], and a relatively new field of computational toxicology has gained popularity [67]. The collective recognition of research questions that require access to harmonized EHS data for analysis and the development of foundational semantic technology makes now the time to bring together the tools and the community.

## 3. Proposed EHS Community Model

**Figure 1:**
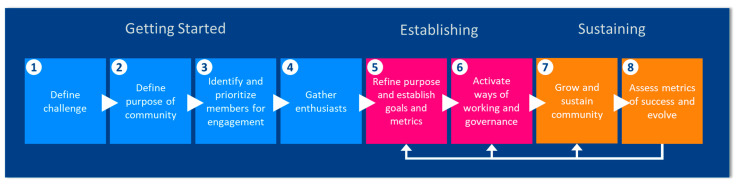
Community development framework. Sources that influenced and guided this framework include [68,69,70,71].

We used the framework shown in Figure 1 to guide the development of the community approach. To initiate this process, the following steps were conducted: a review of prior EHS-related community-building efforts, a scan of research and guidance on collaboration and community building, interviews with successful communities, and working group discussions. From these actions, an approach that comprised a community organization model, a repository of use cases, and community events, was proposed. This approach is anticipated to evolve over the next year through feedback from planned community events.

*Previous workshops*: One of the first workshops to build an EHS language community was hosted by NIEHS in 2014 [59]. The intent of the workshop was to clarify research areas that would be advanced by using EHS language standards, identify stakeholders interested in creating a community to champion standards, and draft guidelines for the development of EHS standards. The workshop participants proposed eight guiding principles for establishing a community: engage a broad community, facilitate collaboration, enable the navigation of existing standards, support the citation and attribution of standards, adopt software development best practices, assist funding early-stage development, create a sustainable and flexible framework, and capitalize on opportunistic development. The formal workshop recommendations included federal funding to ensure the development, expansion, and adoption of standards, as well as a phased approach to development. The first phase addressed the need to identify relevant EHS research questions and use cases for the immediate application of semantic standards. Phase two suggested the development of a web-based standards toolkit to enable easier navigation of existing standards, the extraction of terms for specific project needs, and contribute terms to expand the standard. The community raised the need for a governance and sustainability plan.

A Computable Exposures Workshop was held in 2020 to “foster the development of data reporting standards and a computational model which will facilitate the inclusion of exposure data in computational analysis of human data” [60]. Four important gaps were identified at the workshop that prevented the adoption and use of computable exposures:

- A minimum reporting standard for exposure science and toxicology.
- Curated mappings across chemical authorities.
- A semantic model for exposure data.
- Ontological coverage.

Participants in this workshop developed use cases and competency questions to guide infrastructure development to fill these gaps. At the end of the workshop, a semantic model for exposure data was proposed. Attempts to make progress on these gaps are ongoing and will contribute to the proposed community effort.

The level of engagement and the well-defined next steps at these workshops indicate that support exists for the development of a sustained community to advance this work.

*Community building and collaboration literature scan*: To guide the formation of an EHS language community, we reviewed case studies and literature reviews of community-building efforts in EHS and similar fields. A success factor that was stressed by Arnaud et al. in the development of an ontology CoP for the Consultative Group on International Agricultural Research (CGIAR) Platform for Big Data in Agriculture is the necessity of the regular engagement of CoP members across relevant networks in data curation for biological, food and agronomic, and socioeconomics research [72]. In a case study on developing a CoP for scientific programming for life scientists, Stevens et al. indicated that identifying a core group of dedicated individuals and identifying champions who can become leaders were factors contributing to the successful formation of CoPs [73]. Pyrko, Dorfler and Eden [70], in their study of CoPs, emphasized that CoPs foster “thinking together” and that “thinking together” is necessary for CoPs to thrive.

*Interviews with existing communities*: Because community building is challenging, we wanted to learn how sustainable and impactful communities have successfully overcome these challenges and what lessons they learned along the way. For this purpose, interviews were conducted with participants that were familiar with the origin story of several communities: Earth Sciences Information Partnership (ESIP), Adverse Outcome Pathway Wiki (AOP Wiki), RDA, and the Open Biological and Biomedical Ontologies (OBO) Foundry. The goals were to learn why and how these communities formed and to understand the essential components or actions that were needed to form, grow, and sustain an effective EHS language community. As a result of these interviews, we distilled several takeaways for creating and sustaining a successful community:

- Form the community around a defined/shared purpose. The community needs to identify its purpose and have a clear understanding of its goals.
- Start with a small circle of champions who can communicate the value of the community.
- Have committed/dedicated financial, technical, and labor resources. Successful communities have an infrastructure to support administrative operations.
- Create a sense of “I found my people” among the members.
- Target a specific action to undertake and grow from there.
- Identify the incentives that are needed to get people actively engaged. While the most likely incentive is that the community activities align with the person’s work-related tasks, some members are simply motivated to make a difference.
- Activate ways of working that meet the community’s culture (e.g., formal versus informal governance, preferred channels of communication).

### 3.1. Proposed Community Organization

This section describes the name, mission, goals, and structure of the proposed community. These are being put forward as starting points for community discussion at the upcoming pre-workshop and workshop events.

The proposed vision of the Environmental Health Language Collaborative is to leverage community-driven environmental health language standards to catalyze knowledge-driven discovery and improve public health.

The mission of the Collaborative would be to advance integrative environmental health sciences research by developing and promoting the adoption of a harmonized language.

To achieve this mission, the community would:

- Define use cases for applying knowledge organization systems in research.
- Foster community-based development of harmonized vocabularies, terminologies, and ontologies.
- Promote and develop methods and tools for applying harmonized language in research.
- Cultivate a vocabulary-aware environmental health community through training and education.
- Apply language standards and best practices for accurate environmental health data and knowledge representation

To implement this mission, the community would comprise three elements:

Community of practice: A community of practice provides a hub to exchange information, ideas, and expertise, as well as advance the appreciation for and adoption of semantic and language approaches through education and training.

Forum for coordination and collaboration: The community serves as a forum to coordinate harmonization activities and collaborate on defining use cases and gaps, prioritizing activities, and developing the language strategies or approaches that are needed for enabling data querying, sharing, and interoperability.

Platform to develop and implement: Based on the identified gaps in the use cases, the community serves to support and promote the development and application of harmonized language solutions to address the use cases’ needs.

**Figure 2:**
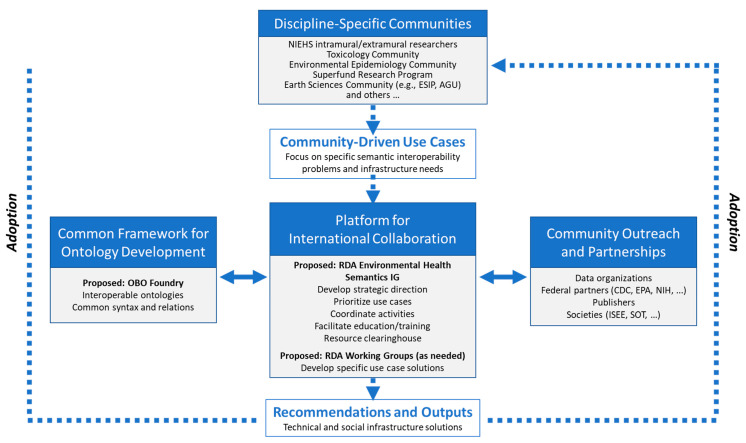
Proposed community model.

Based on interviews with organizations and discussions within a community model working group, the community model shown in Figure 2 was proposed. A key aspect of any community is having an infrastructure for communications, hosting meetings, and other daily operational activities. One of the recurring messages from stakeholders and community interviews was to not “reinvent the wheel.” As such, the Research Data Alliance (RDA) was proposed to provide structure for the EHS community.

The RDA started in 2013 with funding from the European Commission, National Science Foundation, National Institute of Standards and Technology, and Australia’s Department of Innovation, with the mission to “build the social and technical bridges to enable open sharing and reuse of data to accelerate data-driven innovation.” It is a community-driven, grassroots organization with more than 11,000 members from 145 countries. Individual membership is free. Individuals come from a variety of disciplines and professions, including researchers, IT architects, project managers, data scientists, publishers, and librarians. Through interest groups and working groups, members exchange knowledge and share discoveries, discuss barriers and potential solutions, define policies, and seek to harmonize standards to enhance/facilitate global data sharing and re-use. The goals and activities of the RDA align with the proposed EHS community mission, and participation within RDA provides an opportunity to tap into international expertise and perspectives from other related disciplines [74,75].

The proposed model begins with individuals and/or groups from discipline-specific communities that generate use cases based on research questions that are of interest to them. These use cases represent the need for harmonized language solutions that will enhance the findability, sharing, and interoperability of EHS data. The use cases will be brought to a proposed RDA Environmental Health Language Interest Group (IG). This IG will provide a platform for the overall coordination and collaboration between interested members. Its goal is to design a strategic direction for developing and adopting semantic solutions, identify and prioritize use cases, coordinate activities, and be a CoP for exchanging information, offering a resource clearinghouse, and fostering education/training. An RDA Working Group could be formed whenever a specific work product needs to be developed. If the product is an ontology, then ideally, its development would follow the OBO Foundry framework to be interoperable with other ontologies.

The IG and WG(s) will work in concert with other relevant communities or partner organizations toward the development and implementation of any recommendations and outputs. Those products will be communicated back to the discipline-specific communities with the anticipation of adoption.

**Figure 3:**
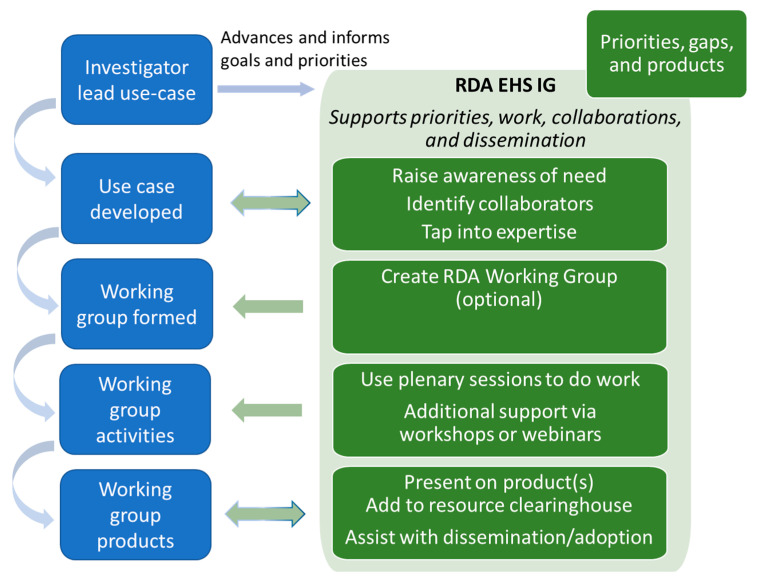
The community model in practice.

*How would this model work in practice*? The intent of the model is to provide support to those developing and applying semantic approaches, as outlined in Figure 3. The example begins with an investigator (or someone else) who has a use case that can benefit from a semantic solution. At this stage, the investigator can work with the RDA Interest Group to raise awareness of the needs, tap into expertise, and identify potential collaborators to work on a team. Use case leads may choose to form a working group outside of the RDA, but they can also decide that creating an RDA working group will assist in gaining broader community input and perspectives. Whether the activities are done within or outside an RDA WG, the IG can support the working group’s activities by offering time at the IG’s plenary sessions to do work and/or provide additional support in the form of workshop activities, presentation time, and webinars. Any developed product(s) from the working group would be brought to the RDA and shared with the broader community, as well as added to a resource clearinghouse. In addition, the RDA IG can assist with disseminating and promoting the adoption of the product if needed. The RDA IG will maintain the catalog of existing use cases, which will aid others in recognizing and prioritizing gaps and issues to which they can provide solutions.

**Figure 4:**
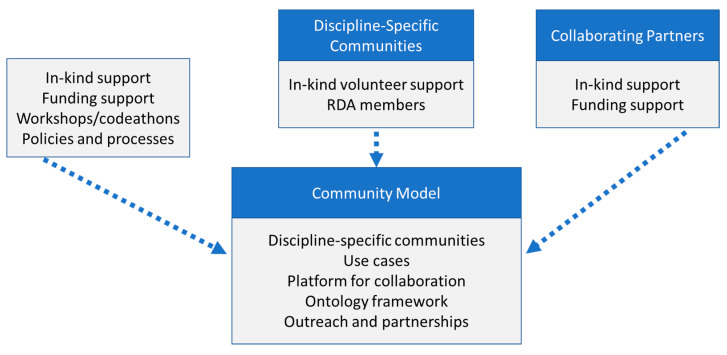
Sustaining the community.

Sustaining the proposed community model requires three supporting players, as shown in Figure 4. The NIEHS proposes to engage by providing in-kind volunteer support to the IG and WGs and working to develop funding strategies for relevant efforts. The NIEHS will help support workshops or other events, such as codeathons, as well as develop policies and processes based on the RDA or other’s recommendations that would advance the community’s goals. In-kind volunteer support will be needed from discipline-specific communities, primarily through serving on the IG and WG. Finally, collaborating partners in academic, federal, and industry sectors will be identified and involved to provide both in-kind contributions, support for funding community activities, and promoting the adoption of outputs.

### 3.2. Community Events

**Figure 5:**
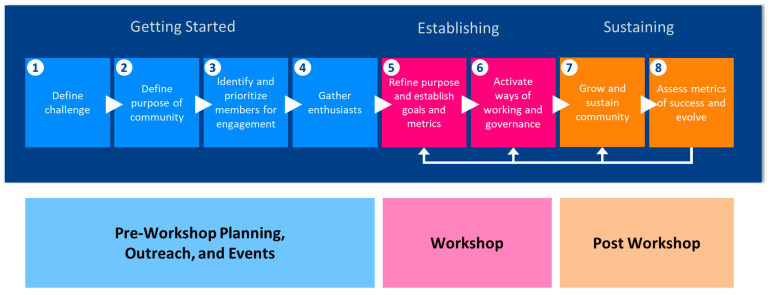
Components of building a community.

The community effort (Figure 5) will launch with a virtual workshop entitled *Catalyzing Knowledge-Driven Discovery in Environmental Health Sciences through a Harmonized Language* on 9–10 September 2021 and will consist of two tracks. The first track will focus on “Build a Sustainable Community” with the goal of achieving agreement on the community’s mission and goals and the development of a roadmap for governance, outreach, and partnerships. The second track, “Develop Solutions,” will be an interactive workathon that will be dedicated to initiating the development of semantic solutions for specific use cases. Participants in this session will define draft use cases in detail and propose and discuss solutions. The desired outputs from this session include (near) complete use case packages that describe the research question, available resources to address the use case, and specified gaps that require solutions. Some use cases may be at the stage of outlining the next steps for developing solutions. We aspire to have working groups form to continue work on the use cases post workshop. A workshop paper will be developed that details the workshop activities, outcomes, and next steps.

In advance of the workshop, we held several pre-workshop events (see Table 1) to provide background on the initiative, allow researchers to learn more about ontologies (e.g., what they are, how to find them, how to apply them), and begin work on pre-defined use cases that will inform workshop sessions.

### 3.3. Use Cases

The authors established a working group of EHS researchers and program officers in 2020 to develop an initial set of draft use cases. These initial use cases were placed into five high-level use case categories with the original use cases serving as example sub-cases. In several instances, the use cases require not only advances in standardized vocabularies but also in statistical and modeling approaches, which represent opportunities to engage with those communities. Although use cases overlap and some consolidation is possible, the use cases are provided in their near original form to avoid errors/simplifications that might result.

Each use case is being championed by a subject matter expert that is familiar with the research question. The champions will be holding small working group meetings to develop a draft use case package (Supplementary File S1) to provide a focus of discussion for the *Develop Solutions* track at the September workshop. The use case package will include a clearer definition of the use case research question, available datasets and ontologies/terminologies that can be used for developing solutions, existing gaps that need to be addressed, and other non-language-related challenges that need to be known.

**Use case #1: What data exists for a given chemical/endpoint/exposure scenario?**

This use case is targeted at finding published data related to a specific set of exposure scenarios. Example sub-cases include:

- What studies measuring endocrine systems perturbation are available?
- What chemicals are chemically similar to compound X and are there any 2-year cancer bioassay data available for these chemicals?
- What animal data exist that provides conclusions on endpoint X given different terms used to describe endpoint X?
- What other data are available for chemical X when it is found in a formulation?
- What assays were “active” for this chemical (where “active” may have different meanings across assays)?

**Use case #2: Combine data from multiple independent studies (e.g., heterogeneous study designs, data collection protocols)?**

This use case is targeted at the issue of integrating data from independent exposure studies. Example sub-cases include:

- Combine individual-level data from multiple independent studies (heterogeneous study designs and data collection protocols) to understand (with increased statistical power) how exposures X and Y impact health outcome Z.
- How can we describe model organism toxicological assays/data in a way that is interoperable and reusable to better understand the phenotypic/epigenomic/transcriptomic impact of exposures X and Y across species A and B?
- Integrate and compare data across labs to support more robust corroboration in the confidence of results from toxicological assessments.
- Given conclusive changes in endpoints to one or more exposures, what other data sources exist on the same exposures and endpoints that can confirm or contradict the findings, including across similar endpoints across different species?
- Given natural text mentions of concepts from scientific studies, what ontology(ies) do these mentions map to in order to normalize terminologies across 100–1000s of studies?

**Use case #3: Given the measures of biological responses to one or more exposures, what are the biological processes that might be related to the observed changes?**

This use case is targeted at supporting the mechanistic interpretations and hypothesis generation that arise given experimental data. Example sub-cases include:

- Given conclusive changes in endpoints to one or more exposures, what are biological processes that might lead to the observed changes?
- How can we use a knowledge graph to fill in the adverse outcome or adverse exposure pathways based on the start or end of the pathway?
- What other modes of action/adverse outcome pathways does this assay hit?
- What assays target this mode of action or key event?
- Given an association between exposure and outcome found in an epidemiological study, find the in vivo and in vitro studies that lend support to the association and that suggest involved bioprocesses, including associations that are dependent on developmental windows.
- Given the signatures of biological responses to exposures from multiple modalities (e.g., gene expression, pathology), can we link these signatures to known biological phenotypes and processes to characterize response signatures and to identify gaps in characterizations?
- Can we link a set of available assays (e.g., in PubChem) to known biological processes and phenotypes in order to better characterize chemical exposures?

**Use case #4: What are the biomarkers, phenotypes, and/or outcomes that can be measured and used as indicators of exposure?**

This use case is targeted at identifying known and hypothesized mechanistic markers of exposures. Example sub-cases include:

- What biomarkers can be used to examine exposure to a given chemical?
- Can we identify biomarkers for different classes of exposures (e.g., exposures to metals/metalloids in soil via dust inhalation, exposure to common pesticides via well water) that are contextualized by delivery route?
- Given conclusive changes in endpoints in response to one or more exposures, what other data sources exist on the same exposures and endpoints that can confirm or contradict the findings, including across similar endpoints across different species?

**Use case #5: What do my unique exposure conditions based on where I live and work (e.g., geographical location, occupation, regulations, hobbies) indicate about potential risks to my health?**

This use case focuses on the linkage of known environmental exposure data to personal health. Example sub-cases include:

- What is my biggest exposure risk based on my geographical location?
- What am I exposed to in my particular line of work? How might this impact my health?
- For what components of X industrial emission do we need more information on health outcomes?
- What levels of exposure to X will decrease the risk of health outcomes?
- What are the health and economic benefits from regulations or policies that reduce exposure to X?
- What are my biggest exposure risks based on work-life conditions, especially where I live and work (work, geography, hobbies)? What is the route of exposure that is most relevant to my specific conditions?
- How does the response to exposure change based on susceptibility (e.g., genetic, disease, SES backgrounds, differences between signatures of exposures, and differences of risk)?

### 3.4. Anticipated Outcomes

The broader biomedical research community is placing increased emphasis on data stewardship and data sharing. For instance, the new NIH Policy on Data Management and Sharing will require NIH-funded researchers to prospectively submit a plan that outlines how data will be managed and shared [76]. Data standards are a key element of these Data Management and Sharing Plans. To be interoperable, (meta)data should use vocabularies that follow the FAIR principles [77]. Community-endorsed data standards and vocabularies are key but are currently a major gap area for EHS. The proposed effort provides a much-needed vehicle for community engagement to address these documented needs.

Previous EHS experience and input from parallel communities emphasize the importance of sustained engagement to move forward. Leveraging the existing international infrastructure through RDA meets this need. Using the RDA model as a guide, some of the anticipated outcomes from these efforts include data and metadata models, roadmaps, extensions to existing EHS-relevant ontologies, and developing new EHS-relevant semantic approaches that will facilitate integration across heterogenous data and nomenclature. This will not only foster data harmonization efforts but improve statistical power and the ability to generate hypotheses more efficiently. The products, outcomes, and recommendations endorsed by this community are expected to enhance data collection and management efforts for NIEHS and the EHS community, making data more findable and interoperable. These efforts can be fed into complementary efforts, such as the Canadian Urban Environmental Health Research Consortium (CANUE) project (https://canue.ca/about/, accessed on 23 August 2021), whose goals include providing a centralized repository for data and tools to study environmental health outcomes.

## 4. Contribute to the Community

This community is open to anyone interested in advancing environmental health sciences research through the development and adoption of a harmonized language. This community will benefit from diverse perspectives—it needs a range of subject matter experts, skill sets, and roles to be represented.

How can you become engaged in this effort?

- Review the materials from previous workshop events at https://www.niehs.nih.gov/research/programs/ehlc/resources/index.cfm (accessed on 23 August 2021).
- Provide input on the proposed community initiative and use cases at https://www.niehs.nih.gov/research/programs/ehlc/ (accessed on 23 August 2021)
- Sign up for our email distribution list to be informed of future events and join the community of researchers, systems developers, ontologists, and others interested in working together on language standards in the environmental health sciences.

## Figures and Tables

**Table 1:** Events for the Environmental Health Language Collaborative.

Event	Date
The Value of Creating Language and Community in Catalyzing Knowledge-Driven Discovery in Environmental Health Research (virtual)	24 June 2021
A Primer on Using Terminologies, Vocabularies, and Ontologies for Knowledge Organization (virtual)	20 July 2021
Catalyzing Knowledge-Driven Discovery in Environmental Health Sciences through a Harmonized Language (virtual)	9–10 September 2021

## Data Availability

Not applicable.

## Supplementary Materials

The following are available online at https://www.mdpi.com/article/10.3390/ijerph18178985/s1, File S1: Environmental Health Use Case Template.
