# Supplementary material for: Catalyzing Knowledge-Driven Discovery in Environmental Health Sciences through a Community-Driven Harmonized Language
Source: Int J Environ Res Public Health. 2021 Aug 26;18(17):8985. doi: 10.3390/ijerph18178985 (PMC8430534; doi:10.3390/ijerph18178985)
Supplement: Supplementary file 1 [file ijerph-18-08985-s001.zip › ijerph-1311382-supplementary.pdf]

Environmental Health Use Case Template

I. Use Case Description	
**Use Case Name**	*Consider a goal-driven use case name*
**Use Case Identifier**	*A unique identifier for reference*
**Owner**	*The organization leading the use case*
**Point of Contact**	*Name, email address, affiliation if not obvious from email, other relevant details*
**Creation / Revision Date**	*Date use case was created or most recently revised*

II. Use Case Summary

Goal
Briefly describe the goal the use case is intended to satisfy; include how resolving the use case would advance EHS research

Description
Summarize the use case, capturing the essential objectives and important variables or concepts that will require terminologies.

Scope
Identify any known boundaries and the intended scope of the use case

Requirements
State any requirement(s)specific to this use case, e.g., capabilities from a process model that the use case supports, reference identifiers, etc.

Priority
Identify the priority of the use case

Stakeholders
Identify all known stakeholders for the use case

Actors/Interfaces
List actors: people, systems, repositories, sensors, any 'thing' that would 1) act on the system (primary actors) or are 2) acted on by the system (secondary actors). Primary actors invoke the use case and benefit from the result.

III. Usage Scenarios

Provide at least two usage scenarios that flesh out the requirements outlined in the summary, including identification of requirements specific to any envisioned ontology or semantically-driven service or application. Scenarios should be described as narrative, with supporting diagrams as appropriate.

IV. Competency Questions

Provide at least 2 competency questions that you will ask of the vocabulary/ontology/ knowledgebase to implement this use case and test the adequacy of the language standard(s).

V. Resources – Knowledgebases, Repositories, Data Sources

In order to support the capabilities described in this Use Case, a set of resources must be available and/or configured.

Data Source Name
Name of dataset or repository

Description
Short description of the dataset and how it was generated

Type
Indicate the type of data; e.g., metabolomic, exposure

Format/Characteristics
Describe the format of the data and use of any standardized identifiers, vocabularies, or other data standards

Sample Data
For each data type, provide an example and highlight components that conform to standards

Owner
Name of resource owner

Link
Link or instructions on access

Access Policies/Usage
Indicate if licensing, PII, or other issues impact access and usage of the data

VI. Resources – Ontologies, Vocabularies, or other Model Services

Resource Name
Name of ontology, vocabulary, or model

Description
Overview of the scope and coverage of the language, including which data variables would be covered by the language

Format
Ontology language or syntactic form; e.g., Owl, RDF, ..

Applications
Briefly describe how the ontology/vocabulary has been applied to data, if relevant

Owner
Name of resource owner

Link
Link or instructions on access

Access Policies/Usage
Indicate any requirements for access and usage

VII. Resources – Other Resources, Services, Applications

Resource Name
Name of other resource

Description
Overview of the resource and its application for the use case

Owner
Name of resource owner

Link
Link or instructions on access

Access Policies/Usage
Indicate any requirements for access and usage

VII. Gaps and Challenges

<u>Data</u>: *After review of available data, indicate any potential data gaps or limitations of the data.*

<u>Language</u>*: After review of languages/models, indicate where gaps in terminologies exist, reconciling overlap/duplication of terms, and/or other limitations of using the existing language standards.*

VIII. Reference Materials

List all materials – policy documents, standards, de-facto standards, glossaries, dictionaries and thesauri, taxonomies, and any other reference materials considered relevant to the use case.

IX. Notes

Space to capture content that has no other place to go.
